# Cryptosporidiosis in Iranian Farm Workers and Their Household Members: A Hypothesis about Possible Zoonotic Transmission

**DOI:** 10.1155/2014/405875

**Published:** 2014-04-27

**Authors:** Morteza Izadi, Nematollah Jonaidi-Jafari, Amin Saburi, Hossein Eyni, Mohammad-Reza Rezaiemanesh, Reza Ranjbar

**Affiliations:** ^1^Health Research Center, Baqiyatallah University of Medical Sciences, Mollasadra Street, Vanak Square, Tehran 14359151371, Iran; ^2^Chemical Injuries Research Center, Baqiyatallah University of Medical Sciences, Tehran 14359151371, Iran; ^3^Isfahan University of Medical Sciences, Isfahan, Iran; ^4^Molecular Biology Research Center, Baqiyatallah University of Medical Sciences, Tehran 14359151371, Iran

## Abstract

*Objectives*. The prevalence of *Cryptosporidium* and the risk factors of zoonotic transmission in Najafabad, Isfahan, Iran dairy farms were examined. *Methods*. One fecal sample was collected from all calves less than 6 months old in eight dairy farms around Najafabad (Isfahan province, Central Iran) as well as individuals working in these farms and their household members. A two-step nested PCR protocol was used to amplify the 18S rRNA gene (830 bp). *Results*. *Cryptosporidium* was identified in the stool of 36 (prevalence 8.5%) of 96 farm workers and 326 household members. Furthermore, 31 (14.2%) of 218 calf samples were positive. *Cryptosporidium parvum* was identified in 15 (72%) of the positive farm workers and 10 (65%) of the positive household members. Of the positive calves, 20 (64.5%) were infected with *C. parvum*, indicating possible zoonotic transmission in these farms. Contact with calves (*P* < 0.0001) was the most significant risk factor of *C. parvum* infection. A considerable negative association was observed between *C. parvum* infection and cleaning of shoes/boots after daily work (*P* = 0.004), hand washing (*P* = 0.013), and use of piped water (*P* < 0.006). In the multivariate analysis with logistic regression, only contact with calves was significant. *Conclusion*. Zoonotic transmission of *C. parvum* due to contact with calves is predominant among farm workers and their household members of this region and appropriate health measures must be applied to control the infection and decrease of zoonotic transmission of this parasite.

## 1. Introduction

*Cryptosporidium* species are apicomplexan protozoa that infect humans and a wide variety of vertebrate animals. Cryptosporidiosis is a self-limiting disease of intestinal tract in immune-competent individuals and a severe chronic disease in immunocompromised patients [1]. Cryptosporidiosis has been considered to be a zoonotic disease and therefore is of potential significance from both disease and public health prospective. Transmission of* Cryptosporidium *spp. from farm animals to humans has been documented in accidental infection of veterinary workers [2, 3]. Cattle have been considered as a substantial source of zoonotic cryptosporidiosis. Young animals appear to be more susceptible to infection and disease, while infections in adults are often asymptomatic or do not occur [4]. Among cattle, calves are prone to infection immediately after birth and continue so for several months [5]. Contact with infected calves has been implicated as the reason of many small cryptosporidiosis outbreaks in humans [6, 7].

Therefore a cross-sectional study was performed to determine the occurrence and prevalence of infection in farm workers and their household members as well as calves to estimate the risk of zoonotic transmission and survey potential risk factors of infection with* Cryptosporidium* zoonotic species.

## 2. Materials and Methods

### 2.1. Sampling and Specimen Processing

One fecal sample was collected from all calves less than 6 months old in 8 dairy farms around Najafabad (Isfahan province, Central Iran) as well as individuals working in these farms and their household members. From September 2009 to March 2010, 218 and 422 fecal samples were collected from calves and humans, respectively. All farm workers and their families agreed to cooperate in this study. Each specimen was placed in a plastic vial, brought immediately to laboratory, and stored at 4°C until analysis.

Age and sex, number of household members, contact with calves, and source of drinking water, hand washing after defecation and daily work, and cleaning shoes/boots after daily works were recorded. The fecal specimens were classified according to their consistency as diarrheic or nondiarrheic.

The fecal specimens were concentrated using a sucrose solution with a specific gravity of 1.200 at a centrifuge speed of 800 ×g for 10 minutes. All of the samples were stained by the modified Ziehl-Neelsen method [8] and examined under bright field microscopy. A sample considered* Cryptosporidium* positive if typical oocysts 4–6 *μ*m in diameter were visible.

### 2.2. DNA Extraction

Microscopically positive fecal samples were subjected to six cycles of freeze-thaw in liquid nitrogen and a 95°C water bath to rupture the oocysts. DNA was isolated from aliquots of frozen stool using the QIAamp DNA stool minikit (QIAGEN, USA) according to the manufacturer's instructions.

### 2.3. 18S rDNA Gene Amplification and Sequencing

A two-step nested PCR protocol was used to amplify the 18S rRNA gene (830 bp). The fragment of the 18S rRNA gene was amplified by PCR using the following primers previously described by Xiao et al. [9]: 5-TTCTAGAGCTAATACATGCG-3 and 5-CCCATTTCCTTCGAAACAGGA-3 for primary PCR and 5-GGAAGGGTTGTATTTATTAGATAAAG-3 and 5-AAGGAGTAAGGAACAACCTCCA-3 for secondary PCR. For the primary PCR step, PCR mixture contained 1x PCR buffer, 3 mM MgCl_2_, 0.2 mM of each dNTP, 2.5 U Taq, 2.5 µL of BSA (0.1 g/10 mL), for each forward and reverse primer in a total of 50 µL reaction volume. A total of 35 cycles, each consisting of 94°C for 45 s, 59°C for 45 s, and 72°C for 1 min, were performed; an initial hot start at 94°C for 3 min and a final extension step at 72°C for 7 min were also included. For the secondary PCR step, the PCR mixture was identical except that a concentration of 1.5 mM MgCl_2_ was used. A total of 40 cycles, each consisting of 94°C for 30 s, 58°C for 90 s, and 72°C for 2 min, were performed; an initial hot start at 94°C for 3 min and a final extension step at 72°C for 7 min were also included. PCR products were analyzed on 1% agarose gel and visualized by ethidium bromide staining.

PCR products were purified and sequenced subsequently using the terminator V3.1 cycle sequencing kit (Applied Biosystems). Sequences were assembled using the program SeqMan (DNASTAR, USA).

### 2.4. Statistical Analysis

The prevalence of* Cryptosporidium* infection and prevalence of* Cryptosporidium* species in preweaned calves were compared with postweaned calves as well as humans. Determination of the association between infection and age, sex, and fecal consistency was performed using chi-square or Fisher's exact test. Results were considered to be significant at *P* < 0.05.

Farmers were asked to complete a questionnaire on potential risk factors for* Cryptosporidium* infection including age, sex, contact with calves, cleaning shoes/boots after daily work, hand washing after work, and contact with soil and use of piped water as their water source.

A univariate analysis for the relation between* Cryptosporidium* infection and potential risk factors was performed (odds ratio) and the significance of variable association was tested using Wald's test. To appraise efficiently the association between exposure and consequence and to assess the effect of possible confounding variables, a multivariate analysis was performed by means of a logistic regression model. Only variables that showed Wald's *P* value smaller than 0.05 were included in the multivariate model and a backward deletion process was then performed. Analysis was carried out using computer software SPSS ver.12 (SPSS Inc., USA). In both the univariate and multivariate analyses, associations were considered significant at *P* < 0.05.

## 3. Results

**Figure 1:**
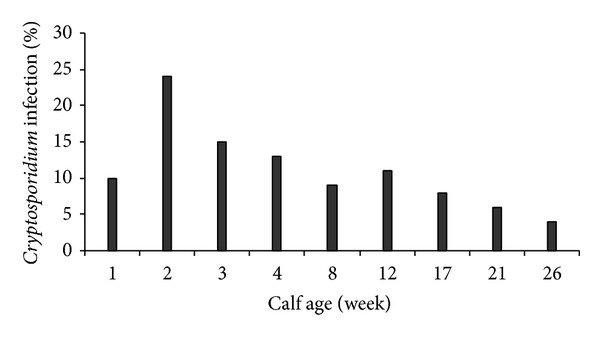
Distribution of* Cryptosporidium* infection in various age groups of calves.

Two hundred and eighteen calf samples were examined and a prevalence of 14.2% (31/218) was found. 20 (21%) of positive samples were detected in preweaned calves, while only 11 (9%) of positive cases were observed in postweaned calves (*P* < 0.05) (Table 1). The highest prevalence was observed in calves with approximately 2 weeks of age (Figure 1). 19 (68%)* Cryptosporidium* positive calves were diarrheic while 12 (32%) were nondiarrheic (*P* < 0.02) (Table 2).

Of 422 humans sampled, 110 were farm workers and 312 were household members. Although farm workers were two times more likely to be infected, the prevalence of* Cryptosporidium *infection was not significantly higher in the former (*P* < 0.105). All 627 samples from calves and humans of known fecal consistency were used to determine relationship between* Cryptosporidium* infection and diarrhea (Table 2).

Total prevalence of* Cryptosporidium* infection in humans was 8.5% (63/422). The infection rate in <5-year-old group and in >5-year-old group was 7 (9.6%) and 29 (8.3%), respectively (Table 3). All positive samples were genotyped.* Cryptosporidium parvum* was the most common genotype identified in 64.6% calves (20/31), followed by* Cryptosporidium bovis* in 29% (9/31) and* Cryptosporidium ryanae* (previously identified as the* Cryptosporidium* deer-like genotype) in 6.4% (2/31) of the calves (Table 1). In preweaned calves* Cryptosporidium parvum *and* Cryptosporidium bovis* were responsible for 18 (90%) and 2 (10%) of infections, respectively. In postweaned calves no* Cryptosporidium parvum *was detected, while* Cryptosporidium bovis* and* Cryptosporidium ryanae* were present in 7 (60%) and 4 (40%) of infections, respectively (Table 1). The results of* Cryptosporidium* and* Cryptosporidium *species prevalence of infection in various locations and herds, based on age and using 18s rRNA amplification and sequencing, were shown in Table 4.

Only 36/63 (57%) microscopy-positive human samples were sequenced. In thirty-six positive human samples* Cryptosporidium parvum* was identified in 28 (78%) samples and* Cryptosporidium hominis* in 8 (22%) samples (Table 3). 19 (68%) of the* Cryptosporidium*-positive calf samples were diarrheic, while only 13 (7%) of the* Cryptosporidium*-negative samples were diarrheic (*P* < 0.002). In humans, 9 (25%) of the positive samples were diarrheic while 27 (75%) of the negative samples were found to be diarrheic (*P* < 0.03). Thus, it was obvious that* Cryptosporidium* infection appears to be associated with the occurrence of diarrhea in calves and humans.

The results of the univariate analysis are shown in Table 5. In humans,* Cryptosporidium parvum* infection was not associated with age less than 5 years (OR = 1.65; 95% CI = 0.67–4.05; *P* = 0.265), contact with soil (OR = 1.3; 95% CI = 0.57–2.97; *P* = 0.527), diarrhea (OR = 1.65; 95% CI = 0.54–5.04; *P* = 0.369), and crowding (OR = 2.06; 95% CI = 0.81–5.21; *P* = 0.117). A significant association was found with contact with calves (OR = 6.68; 95% CI = 1.99–22.39; *P* < 0.0001). Sex was not correlated with* Cryptosporidium parvum* infection (OR = 1.06; 95% CI = 0.49–2.3; *P* = 0.877).

Cleaning of shoes/boots after daily work (OR = 0.33; 95% CI = 0.15–0.73; *P* < 0.004), use of piped water (OR = 0.34; 95% CI = 0.15–0.75; *P* < 0.006), and hand washing after daily work and defecation (OR = 0.38; 95% CI = 0.17–0.83; *P* < 0.013) were protective factors against* Cryptosporidium parvum* infection.

In multivariate analysis with a backward deletion process, the nonsignificant factors were excluded from the model. In the final analysis only contact with calves maintained a significant association with infection (Table 6).

## 4. Discussion

In this study we examined fecal samples of calves and farm workers and their household members to determine the prevalence of* Cryptosporidium* infection and* Cryptosporidium* species in calves and humans to assess zoonotic transmission possibility between calves and humans.

Several studies evaluated the prevalence of* Cryptosporidium* infection in bovine and humans in Iran [10–12]. In our study the prevalence of* Cryptosporidium* infection was 14.2% (31/218) and 8.5% (36/422) in calves and humans, respectively. Azami et al. found 6.2% (30/480) prevalence of infection in calves and cattle in Isfahan [10]. The higher prevalence in this study also in Isfahan may be due to the use of highly sensitive 18s rRNA gene amplification. Moreover, the cattle ages might have been different with younger ones being more likely to be infected. Also, the target population which was studied in two studies was different (children and immunocompromized patients versus normal adults). Previously, prevalence was reported to be 12% (35/292), 17.7% (19/107), 18.8% (51/275), and 6.4% (31/482) in calves of various regions of Iran, respectively [11–14]. An infection rate of 19.5% of* C. parvum* among 174 preweaned calves was significantly higher than the 3.1% among 98 postweaned calves (*P* < 0.0006) [13]. Similar results have been observed in calves in point prevalence studies worldwide [15–19]. The highest prevalence was found in calves by two weeks of age (24%) which is consistent with reports from countries throughout the world [15, 19, 20]. Molecular characterization of* Cryptosporidium* using 18s rRNA genes indicated the presence of* C. parvum*,* C. bovis,* and* C. ryanae* in agreement with other point prevalence studies of calves [21–23]. In the present study,* C. parvum* was responsible for 64.5% while* C. bovis *and* C. ryanae* were identified in 29% and 6.5% of infections of calves, respectively. The most prevalent genotype in preweaned calves was* C. parvum* (91%) followed by* C. bovis* (9%). Otherwise in postweaned calves most infections were due to* C. bovis* (78%),* C. ryanae* was responsible for 22% of infections, and no animal with positive* C. parvum* specimen was observed in this group. It had been reported that cattle are commonly infected with three other* Cryptosporidium *spp. in addition to* C. parvum*, including* C. bovis*,* C. ryanae* (previously known as* Cryptosporidium* deer-like genotype), and* C. andersoni*. Numerous recent studies in various areas indicated the existence of an age-related occurrence of* Cryptosporidium *spp. In dairy cattle,* C. parvum* is mostly found in preweaned calves,* C. bovis* and* C. ryanae* in postweaned calves, and* C. andersoni* in yearling and adult cattle [21, 24–27]. Thus, in cattle, only preweaned calves are major source of* C. parvum* (one of the most prevalent zoonotic genotypes) [28].

In 36 PCR-positive human samples* C. parvum* was identified in 28 samples (78%) and* C. hominis* (nonzoonotic genotype) in 8 samples (22%). As shown earlier, the distribution of* C. parvum* and* C. hominis* in humans differs in different geographic regions. In European countries, both* C. parvum* and* C. hominis* are common in humans [29, 30]. In the Middle East especially in Iran,* C. parvum* is the dominant species in humans [11, 31–33]. In the rest of the world,* C. hominis* is usually the predominant species in humans [34–36].* C. parvum* was identified in 72% of the positive farm workers and in 65% of the household members. These results are consistent with Siwila et al.'s study in Zambia that found* C. parvum* in 75% of positive farm workers and 60% of the household members [37]. All seven positive samples from less than 5-year-old children were identified as* C. parvum*, while in twenty-nine positive samples of more than 5-year-old individuals, 21 samples (72.4%) were* C. parvum* and 8 samples (27.4%) were* C. hominis*.

There are age-association variations in the disease burden between* C. parvum* and* C. hominis* [29]. In Iran, Kuwait, and Turkey almost all cryptosporidiosis cases in children are caused by* C. parvum*, while in The Netherlands and UK* C. hominis *was more commonly found in children [29, 38]. This study is the first survey of* C. parvum* potential risk factors in Iranian farm workers and their household members. We found significant association between contact with calves and* C. parvum* infection (OR = 6.68; 95% CI = 1.99–22.39; *P* < 0.0001). Robertson et al. reported calf contact away from home as the risk factor of* C. parvum* infection in Melbourne (OR = 2.9; 95% CI = 1.5–5.7) and Adelaide (OR = 5.1; 95% CI = 1.5–17.3) [39]. Roy et al. reported contact with calves or cows (OR = 3.5; 95% CI = 1.8–6.8; *P* < 0.01) as risk factor in USA [40]. Hunter et al. identified touching cattle (OR = 3.9; 95% CI = 1.4–10.0) as risk factor of infection in Wales and New England [41]. There is no significant association between age, sex, crowding, diarrhea, and contact with soil and* C. parvum* infection. But there was significant negative association between cleaning of shoes/boots after daily work, hand washing after work and defecation, and use of piped water and* C. parvum* infection. In other words these factors were potentially protective against* C. parvum* infection. In multivariate analysis, all risk factors for* C. parvum* infection were excluded except contact with calves. In this study contact with calves was the most important risk factor of* C. parvum *infection of farm workers and their household members, both in univariate and multivariate analyses. Cattle have been considered to be an important source of zoonotic cryptosporidiosis since the 1980s. It seems that the most important cause of many small cryptosporidiosis outbreaks in veterinary students and veterinarians, research technicians, and children attending agricultural camps and fairs is contact with infected calves [42–44]. But nowadays, there are many reports of different types of* Cryptosporidium* infections in different parts of the world every day which supports a powerful and efficient transmission [45].

The finding that 75% of the* Cryptosporidium*-positive humans were asymptomatic (nondiarrhoeic) is surprising and noteworthy. It is worth discussing this as it probably represents a further risk factor for transmission especially if this was shown to be in young children from which transmission is likely to occur. Although prevalence of asymptomatic careers in this study was reported as high as 75%, this range may be variable by species of* Cryptosporidium*, the general health condition of human host for example being immunocompromised such as HIV positive and kidney transplant patients, the prevalence of positive calves and animal and previous outbreaks in that geographical region [46–49].

## 5. Conclusion 

The results of present study clearly show that humans working close to calves are more likely to be at risk of zoonotic infection with* C. parvum*. As reported earlier, the clear predominance of* C. parvum* in Iranian people might consider the result of zoonotic transmission [31]. However, more comprehensive studies in* Cryptosporidium* spp. are needed to clarify accurately the zoonotic transmission of* Cryptosporidium* zoonotic genotypes. Particularly, further subtyping of* C. parvum* in humans and animals is needed to improve our knowledge of routes of transmission and potential risk factors in this region and other locations of Iran.

## Figures and Tables

**Table 1:** Prevalence of *Cryptosporidium* species in calves.

Age	*n*	*Cryptosporidium *		*C.parvum *		*C.bovis *		*C.ryanae *	
		Pos.	Prevalence (%)	Pos.	Prevalence (%)	Pos.	Prevalence (%)	Pos.	Prevalence (%)
Preweaned	94	20	21	18	90	2	10	0	0
Postweaned	124	11	9	0	0	7	60	4	40

Total	218	31	14	20	62.5	9	25	4	12.5

**Table 2:** * Cryptosporidum* prevalence in diarrheic and nondiarrheic calves and humans.

Host	*Cryptosporidium *	Sample number	Diarrheic* (%)	Nondiarrheic (%)
Calve	Pos.	31	19 (68)	12 (32)
	Neg.	187	13 (7)	174 (93)
Human	Pos.	36	9 (25)	27 (75)
	Neg.	386	31 (8)	355 (92)

*Diarrhea was defined as having ≥1 day with >3 liquid or semiliquid stools.

**Table 3:** Prevalence of *Cryptosporidium* and *Cryptosporidium * genotypes in the farm workers and their household members based on age.

Age	*n*	*Cryptosporidium *		*C.parvum *		*C.hominis *	
		Pos	Prevalence (%)	Pos.	Prevalence (%)	Pos.	Prevalence (%)
<5 years	73	7	9.6	7	100	0	0
>5 years	349	29	8.3	21	72.4	8	27.6

Total	422	36	8.5	28	78	8	22

**Table 4:** Prevalence of *Cryptosporidium* and *Cryptosporidium* genotypes in the calves based on age.

Location	Herd	Age	*n*	*Cryptosporidium *		*C.parvum *		*C.bovis *		*C.ryanae *	
				Pos.	Prevalence (%)	Pos.	Prevalence (%)	Pos.	Prevalence (%)	Pos.	Prevalence (%)
Goldasht	G1	<2 months	25	3	12	3	100	0	0	0	0
		2–6 months	14	1	7.1	0	0	1	100	0	0
	G2	<2 months	22	3	13.6	2	9.1	1	4.5	0	0
		2–6 months	15	2	13.3	0	0	2	100	0	0

Juzdan	J1	<2 months	13	2	15.4	2	100	0	0	0	0
		2–6 months	7	0	0	0	0	0	0	0	0
	J2	2–6 months	14	3	21.4	0	0	2	14.3	1	7.1

Dehegh	D1	<2 months	11	2	18.1	2	100	0	0	0	0
		2–6 months	9	1	11.1	0	0	1	100	0	0
	D2	<2 months	13	2	15.4	2	100	0	0	0	0

Alvijeh	O1	<2 months	33	8	24.2	7	21.2	1	3	0	0
		2–6 months	17	2	11.8	0	0	1	5.9	1	5.9
	O2	<2 months	14	2	14.3	2	100	0	0	0	0
		2–6 months	11	0	0	0	0	0	0	0	0

Total			218	31	14.2	20	9.2	9	4.1	2	0.9

**Table 5:** Univariate analysis of association between potential risk factors and *Cryptosporidium parvum *infection in farm workers and their household members.

Risk factors	Odds ratio	95% CI	*χ* ^2^	*P* value
Age^a^	1.65	0.67–4.05	1.24	0.265
Sex^b^	1.06	0.49–2.30	0.24	0.877
Crowding^c^	2.06	0.81–5.21	2.45	<0.117
Diarrhea	1.65	0.54–5.04	0.8	<0.369
Contact with calves	6.68	1.99–22.39	12.31	0.0001
Cleaning of shoes/boots	0.33	0.15–0.73	8.1	<0.004
Hand washing after daily work and defecation	0.38	0.17–083	6.20	<0.013
Use of piped water	0.34	0.15–0.75	7.55	0.006

^a^<5 years versus >5 years old.

^b^Male versus female.

^c^Crowding is defined as families with more than 4 members.

**Table 6:** Final model of multivariate analysis of *Cryptosporidium parvum* infection risk factors.

Risk factor	Odds ratio	95% CI	Regression coefficient	SE	*P* value
Contact with calves	8.3	2.3–26.5	2.1	0.5	0.0000
